# Effect of Superior Ovarian Nerve and Plexus Nerve Sympathetic Denervation on Ovarian-Derived Infertility Provoked by Estradiol Exposure to Rats

**DOI:** 10.3389/fphys.2019.00349

**Published:** 2019-04-09

**Authors:** Miguel del Campo, Beatriz Piquer, Jason Witherington, Arun Sridhar, Hernan E. Lara

**Affiliations:** ^1^Laboratory of Neurobiochemistry, Faculty of Chemistry and Pharmaceutical Sciences, Centre for Neurochemical Studies in Neuroendocrine Diseases, Universidad de Chile, Santiago, Chile; ^2^Galvani Bioelectronics, Stevenage, United Kingdom

**Keywords:** polycystic ovary syndrome, ovary, denervation, sympathetic nerve, fertility

## Abstract

Sympathetic innervation of the ovary in rodents occurs via two routes: the superior ovarian nerve (SON), which runs along the ovarian ligament, and the plexus nerve (PN), which is mainly associated with the vasculature. SON and ovarian norepinephrine (NE) levels play a major role in regulating ovarian cystic health. Although it was previously described that the polycystic ovarian phenotype (PCO) is causally related to hyperstimulation of the sympathetic nerves of the ovary, much less is known, however, regarding the role of PN in ovarian physiology. We studied the role of SON and PN in relation to the maintenance of the PCO phenotype induced in the rat by exposure to estradiol valerate (EV). EV exposure at 24 days old (juvenile exposure) increases NE in the ovary for up to 90 days after EV injection. SON or PN denervation (SONX and PNX) decreased NE. SONXreversed the acyclic condition from 30 days after surgery (*p* < 0.05), but PNXdid not. SONX was more effective than PNX to downregulate the increased number of cysts induced by EV, with the presence of the corpora lutea (CL, signifying ovulation) in the SONX group. Seventy percent of SONX rats presented with pregnancy at 60 days post-EV (6 of the 7 sperm-positive rats were pregnant); however, SONX rats had a reduced number (half) of pups compared with vehicle-treated rats and 60% more pups than EV rats. These data suggest that the SON plays a predominant role in follicular development, ovulation and pregnancy during ovarian diseases.

## Introduction

Gonadotropins from hypothalamus-pituitary glands regulate ovarian function. However, recent evidence points to neural regulation of the ovary with neurotransmitter release from the sympathetic nerve endings regulating ovarian follicular health (Curry et al., 1985; Lara et al., 1993; Stener-Victorin et al., 2008). Much evidence supports complementary control performed by sympathetic nerves innervating the ovary in addition to the well-known control of gonadotropin in ovary function (Lara et al., 1993, 2002). It has been described that changes in the activity of sympathetic nerves are functionally coupled to androgens but not estrogen secretion from the ovary (Barria et al., 1993). In animal ovarian disease models (induced by either estradiol administration, chronic sympathetic stress or pharmacological β-adrenergic receptor activation), a sustained increase in sympathetic nerve activity has been shown to be causally related to the appearance of a polycystic phenotype at the ovary, with characteristics that resemble, in many aspects, the polycystic ovary syndrome found in women (Barria et al., 1993; Lara et al., 2000; Dorfman et al., 2003; Luna et al., 2012). Sympathetic nerves to the ovary in rodents arrive principally by two routes. First, the superior ovarian nerve (SON) fibers travel through the ovarian ligament and enter the ovary through the hilum to organize around the follicles without penetrating the granulosa cell layer (Lawrence and Burden, 1980). The fibers are associated with steroid secretion and follicular development of the ovary. The second group of nerves, the plexus nerve (PN) is associated with the vasculature, and the fibers enter the ovary through the hilum. It is not known if the PN regulates steroid secretion and follicular development (Lara et al., 2002). The SON has been implicated in the development and maintenance of the hyperandrogenic condition and blunted follicular development associated with the polycystic ovary phenotype (PCOS). Evidence comes from animal models of PCOS where the administration of estradiol valerate (EV) to rats increases norepinephrine (NE) concentration in the ovary and is associated with the development of the polycystic phenotype with absent estrous cycling and reduced corpus lutea. In this model, surgical denervation of the SON decreased ovarian NE, and, intriguingly, the rat recovers estrous cycling activity and increases corpus luteal numbers (Brawer et al., 1986; Barria et al., 1993). These results suggest that neural signaling through the SON regulates ovulatory behavior (Barria et al., 1993; Lara et al., 1993; Morales-Ledesma et al., 2010). Since such information does not exist for PN, we designed two experimental procedures: (1) We blocked the estradiol-induced increase in the sympathetic tone of the ovary by local surgical denervation of the SON (SONX) or PN (PNX) to test whether we can discriminate between nerves to recover follicular development and ovulation after denervation. (2) Since SON is postulated to regulate steroid secretion, we tested the impact of SON on fertility upon mating.

## Materials and Methods

### Study Design

All animal studies were carried out in accordance with the animal protocols approved by the Bioethics Committee of the Faculty of Chemistry and Pharmaceutical Sciences at the Universidad de Chile (UCh). A first protocol with 48 rats was evaluated on October 20, 2015. Code CBE2015-20 and a second group of 40 rats for fertility were amended on April 20, 2016. They also complied with national guidelines (CONICYT Guide for the Care and Use of Laboratory Animals).

#### Animals

We used Sprague-Dawley rats from our facilities. The rats were maintained under a 12 h dark: 12 h light cycle at 21 ± 1°C during all experiments. They were fed with Labdiet rat food (Brentwood, MO, United States), provided with enrichment, and cohoused with two other rats of the same age to maintain normal behavior. All the rats were hemiovariectomized (ULO) during the surgical denervation process (or sham operation) to eliminate a putative effect of the contralateral ovary on the hypothalamus. Some asymmetry was described between the right and left ovary regarding the hypothalamic control of the ovary (Pastelin et al., 2017; Rosas et al., 2018). It was described that each ovary has a different capacity to release steroids; thus, a hormonal signal from one ovary could affect the contralateral ovary by altering the hormonal signals that after arrive to the ovary through the circulation. This was the reason why we decided to do all control (either sham-injected or sham-denervated rats) with ULO to compare in the same conditions all the groups. Rats were injected at 24 (Group 1) or at 14 (Group 2) days old. Both age groups were selected because we previously demonstrated (Cruz et al., 2012) a developmental window in which the administration of a single injection of EV solution produced a permanent effect on the ovary. Next, 24 days of age was selected as the age limit for reversion, and the PCO phenotype was established at the age at which the rats were euthanized. The 14 days old administration of EV was selected as under the limit of reversion and time experiments (similar to fertility studies) can no longer be performed to study fertility. Control rats were injected with sesame oil, and experimental rats were injected with EV in a single intramuscular (i.m.) dose of 10 mg/kg of EV (Sigma Chem Co., MO, United States) dissolved in sesame oil.

The rats were randomly assigned to the following two groups:

**Group 1. Ovary function group** (Bioethical permission code CBE2015-20):

(a) Sham group: Subcutaneous injection of sesame oil at 24 days old. At 54 days old, the right ovary was removed (Sham 30 days) and the left ovary was exposed but not denervated (Sham). One group was euthanized at 84 days old and the other group at 114 days old.
(b) PCOS group: PCOS was induced at 24 days old by a single subcutaneous injection of EV. At 54 days old, the rats were anesthetized; the left ovary was surgically denervated either on the SON (SONX) or, in another group, in the PN (PNX). The right ovary was removed; one half was stored at -80°C for analysis, and the other half was fixed in 10% formaldehyde (EV 30 days group). One group was euthanized at 84 days old and a second group at 114 days old (see Table 1).

**Table 1:** Distribution of the rats to study ovary function.

Ovary function				
**Groups**				
**Age**	**24 days old**	**54 days old**	**84 days old**	**114 days old**

**Procedure**	**EV administration**	**Surgery**	**Euthanasia**	**Euthanasia**
N		30 days	60 days	90 days
12 rats	Sesame oil injected	Sh + ULO	EU (6 rats)	EU (6 rats)
6 rats	EV	SHAM+ULO	EU	
6 rats	EV	SHAM+ULO		EU
6 rats	EV	SONX+ULO	EU	
6 rats	EV	PNX+ULO	EU	
6 rats	EV	SONX+ULO		EU
6 rats	EV	PNX+ULO		EU
48 rats				

**Group 2. Fertility group** (Bioethical permission code CBE2015-20 amended on April 20, 2016):

We used 4 groups of 10 rats each. Due to a possible reversion of the effect of EV at longer times after EV (90 days), the rats used in this experiment were injected at 14 days old. We have shown that rats injected with EV at this age have no reversion of the PCO phenotype (Cruz et al., 2012). Control rats were injected with sesame oil and the EV-treated rats with a single dose of 10 mg/kg of EV (Sigma Chem Co, MO, United States) dissolved in sesame oil. The distribution of the four experimental groups is shown in Table 2.

**Table 2:** Distribution of rats for the fertility study.

Fertility				
**Groups**				
**Age**	**14 days old**	**44 days old**	**74 days old**	**104 days old**

**Procedure**	**EV administration**	**Surgery**		**Fertility test**
N		EV 30d	EV 60d	EV 90d
10 rats	EV	SONX+ULO	fertility test	
				
10 rats	EV	SHAM+ULO		fertility test
				
10 rats	EV	SONX+ULO		fertility test
				
10 rats	SHAM injected	ULO		fertility test
40 rats				

Our primary outcome measures were evaluated in the animals from the experimental groups:

• Estrous cycle phase, evaluated daily by vaginal cytology
• Ovarian morphological phenotype, evaluated at all ages
• Ovarian NE concentration, evaluated at all ages
• Fertility capacity, evaluated in Group 2 at 75 and 105 days old (Group 2)

#### Processing of Tissues:

##### Estrous cycling activity

Estrous cycling activity was checked daily from puberty up to the end of the procedure including the fertility test. Vaginal fluid was obtained daily using a plastic pipette, placed on the glass slide and observed under a light microscope. Epithelial cells (large, round, nucleated), cornified cells (irregular, without nucleus), and leukocytes (small, round) were identified, and their proportion was analyzed to define proestrus (P) during the night of ovulation (Dissen et al., 1996), estrus (E) (the day after ovulation) and diestrus (D) (the two previous days before P), as previously described (Marcondes et al., 2002).

Because ULO and denervation were performed during a single surgery at 45 days old, the extracted right ovary was halved and one half was used to determine the ovarian NE content to check for increased NE levels after 30 days of EV administration (Lara et al., 1993). The other half was fixed in formaldehyde, cut in 6 μm slices, stained in hematoxylin-eosin and analyzed for the presence of precystic structures or follicular cyst and corpora lutea in the central slice of the ovary.

##### Mating procedure

Once the rats attained the age of 75 or 105 days old, control and denervated rats were checked for estrous cycling activity to determine the proestrus day. On the night of the proestrus phase (during which ovulation occurs), the rats were mated with males of proven fertility. The following morning, the rats were checked to look for a vaginal sperm plug. It they were positive, the rats were assigned as day 0 of pregnancy. If the rats did not show proestrus (as seen with EV-treated rats) during a 2 weeks period, they were permitted to stay each night for 2 weeks with the males, and they were checked every morning for the sperm plug. If there was no sperm plug after 2 weeks, the rats were assigned as infertile (Urra et al., 2016; Piquer et al., 2017).

##### Number of offspring and implantations

Number of offspring and implantations in uterine horns: During the day of birth, the number of live pups was quantified. Later, the mothers were euthanized on day 4 after delivery, and the uterine horns were exposed to view the number of implants in the uterine horn and compare it with that of offspring born (Urra et al., 2016; Piquer et al., 2017).

##### Analysis of follicular structures

Analysis of follicular structures were analyzed in the central slice. In this slice, the cystic, antral and atretic follicles, as well as the corpora lutea, were quantified. Once the follicle was classified, it was followed to the adjacent slices to determine the total size and mean diameter (in which the nucleus of the oocyte was found). The same procedure was performed for the corpora lutea. We used the following classification: antral follicles were those with more than 3 healthy granulosa cell layers, the antrum and with a clearly visible nucleus of the oocyte. atretic follicles had more than 5% of cells with pyknotic nuclei in the largest cross-section and exhibited shrinkage and an occasional breakdown of the germinal vesicle. Cystic follicles were devoid of oocytes and displayed a large antral cavity, a well-defined thecal cell layer, and a thin (mostly monolayer) granulosa cell compartment containing apparently healthy cells. Corpora lutea presented the largest size in the ovary, absence of oocytes and presence of luteal cells instead of granulosa cells (Lara et al., 2000).

##### Quantification of intraovarian NE levels

Quantification of NE levels was carried out using the competitive NE ELISA kit – Research^®^ (IMMUSMOL, Pessac, France). We followed the instructions of the manufacturer. NE was extracted using a cis-diol-specific affinity gel, acylated and then derivatized enzymatically. The antigen was bound to the solid phase of the microtiter plate. The derivatized standards, controls and samples, as well as the solid-phase-bound analyte, compete for a fixed number of antiserum binding sites. The antibody bound to the solid phase was detected using an anti-rabbit IgG-peroxidase conjugate and TMB as a substrate. The reaction is monitored at 450 nm. The sensitivity was 2 pg/ml, and the intra and interassay variability were 8.4 and 8.0%, respectively. The cross reactivity found was 0.14% for adrenaline and 1.8% for dopamine.

### Statistical Analysis

Differences between the control and treatment groups were analyzed using paired *t*-test or unpaired *t*-test according to the comparison group. For within-animal comparison, the two-tailed *t*-test was used. When groups were analyzed, we used one-way ANOVA with Tukey’s multiple comparisons test. Significance was set at *p* < 0.05. The minimum number of rats to be used for the experiments was calculated as described in Zar (1984). The Shapiro-Wilk test was applied to verify the normal distribution of data. All statistical analyses were performed using GraphPad Prism v5.0 software (GraphPad Software, San Diego, CA, United States).

## Results

### Study on Ovary Function

#### The Effect of Ovary Denervation on the Estradiol-Induced NE Concentration in the Rat Ovary

**FIGURE 1:**
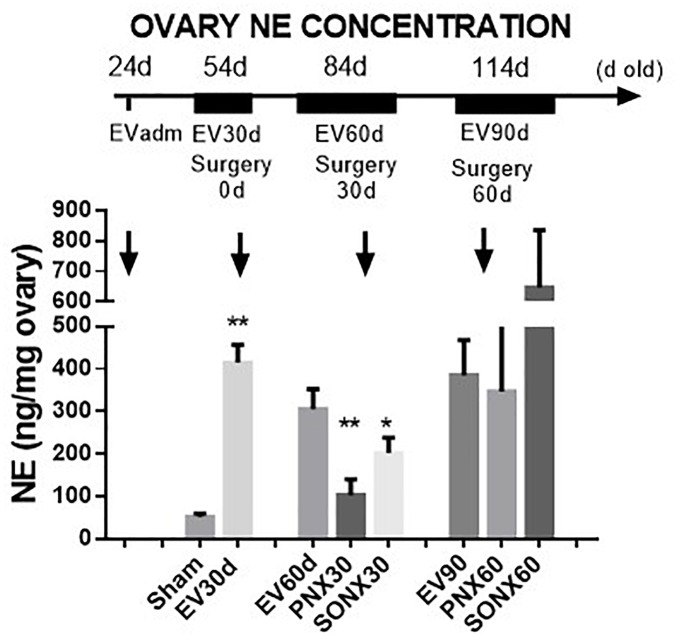
Changes in the NE concentration at different periods of EV treatment. Rats were injected with estradiol valerate at 24 days old. Thirty days later, they were hemiovarectomized, and half of the ovary was used to measure NE. Each group corresponds to six individual rats with the exception of the EV30 day groups that correspond to the total number of ovaries obtained after surgical resection (*n* = 18). The data are presented as the mean ± SEM. *^∗^p* < 0.05 vs. EV and ^∗∗^*p* < 0.01 vs. EV.

Estradiol valerate was administered at 24 days old. After 30 days, the NE concentration was measured in the contralateral ovary obtained during surgical or sham denervation of the left ovary during the process of unilateral ovariectomy. The NE concentration increased 5 times after 30 days of EV administration. The ovaries obtained from the total number of EV-treated rats were used in this determination; thus, the total number of samples corresponded to 18 rats. A similar concentration was found in rats 60 days post-EV injection and remained high until 90 days post-EV injection. PNX and SONX denervation was effective in decreasing the NE concentration after 30 days of surgery, but it was recovered after 60 days post-nerve transection (Figure 1).

#### The Effect of Ovary Denervation on the EV-Induced Changes in the Estrous Cycling Condition in the Rat

**FIGURE 2:**
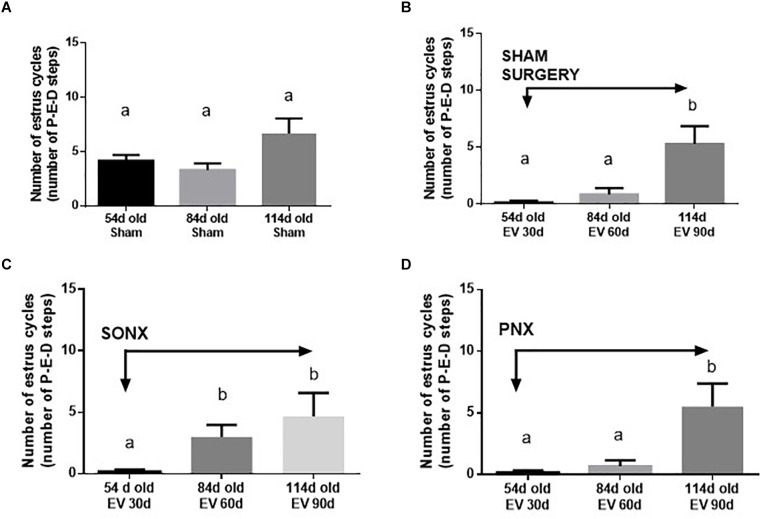
Effect of superior ovarian nerve denervation (SONX) and plexus nerve denervation (PNX) on the estrous cyclicity of rats at different ages (number of estrous cycles: **A**, in sham injected rats; **B**, after sham surgery; **C** at different times after SON denervation) and **D** at different times after PN denervation). Each condition corresponds to the mean number ± SEM of six different rats. Different letters represent significant differences (*p* < 0.05) between the treatment groups.

The estrous cycling activity represents the transition between the different stages of the estrous cycle as determined by vaginal lavage analysis at microscopy each day. The transition from proestrus to estrus implies the occurrence of ovulation, and it is usually used as an index of ovulatory behavior *in vivo* (Marcondes et al., 2002). Figure 2A shows the estrous cycling behavior of Sham-operated rats. Sham rats started with estrus cycling activity at 33.6 ± 2.0 days (the day of vaginal opening). By 54 days of age, they appeared to have approximately 4 estrus cycles, maintaining the number of cycles by 84 days and a small non-statically increase at 114 days old. The latter is the period where Sprague-Dawley rats present with the highest fertility (Cruz et al., 2017). Estradiol-treated rats presented early vaginal opening (29.3 ± 0.8 days vs. 33.6 ± 2.0 days; *p* < 0.001). In contrast to the vehicle group, EV-injected rats presented little to no estrus cycles during this period and remained low after 60 days of EV exposure. A partial recovery was found after 90 days of EV administration (Figure 2B). The effect of SONX denervation is shown in Figure 2C. The EV-30d post-SONX group showed a statistically significant improvement in estrous cycling behavior compared with no denervation (*p* < 0.05), and this was maintained after 60 days of denervation. PNX (Figure 2D) was not effective to recover the estrous cycling activity after 30 days of denervation. No recovery at 60 days of EV and 30 days of denervation was also found in these rats.

#### The Effect of Ovary Denervation on the EV-Induced Polycystic Phenotype in the Rat

**FIGURE 3:**
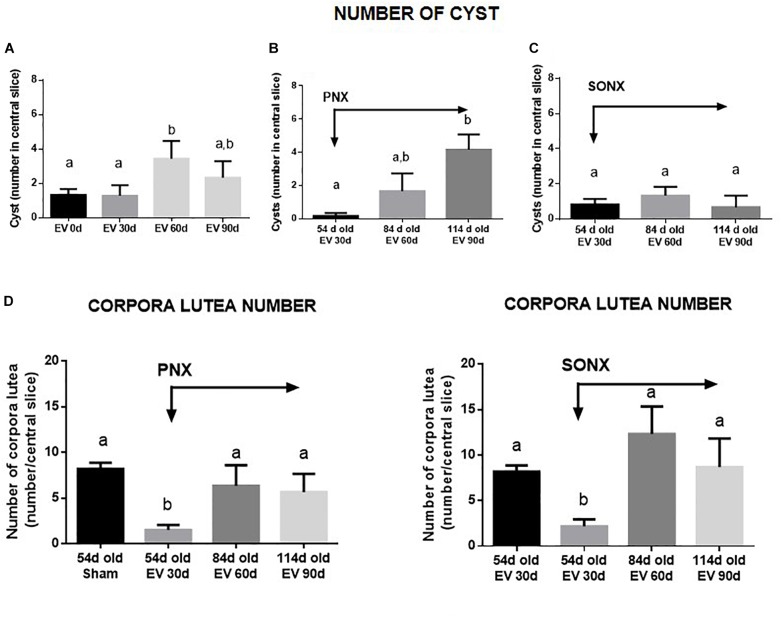
Effect of superior ovarian nerve denervation (SONX) and plexus nerve denervation (PNX) on EV-induced cyst formation (**A**, sham operated-EV injected rats, **B**, EV injected and PNX operated rats and **C**, EV injected and SONX rats) and the corpora lutea number in (**D**, left side: EV injected at different times after PNX. Right side EV injected at different times after SONX) the rat. Each condition corresponds to the mean number ± SEM of six different rats. Different letters represent significant differences (*p* < 0.05) between the treatment groups.

After EV exposure, the PCO phenotype was established at 60 days post-EV administration (Brawer et al., 1986). The number of cysts increased at 60 days after EV (Figure 3A) and was maintained with a slight decrease after 90 days of exposure to EV. PNX did not affect the increased number of cysts found at 30 and 60 days after denervation (Figure 3B). SONX, however, was effective to maintain a low number of cysts up to 60 days of denervation (Figure 3C). Although there is an increase in atretic antral follicles by EV exposure as previously found with the same protocol (Lara et al., 2000), SONX or PNX did not modify the effect of EV on healthy and atretic antral follicles (data not shown). To analyze the effect of denervation on the number of corpora lutea and, hence, on the ovulatory capacity of the rats treated with EV, we quantified the mean number of CLs in the central slice of the ovaries fixed in formaldehyde and stained with hematoxylin/eosin. As shown in Figure 3D, after an initial decrease in the number of CLs, no changes were found in the number of CLs either by PNX or by SONX at 30 or 60 days after denervation.

#### The Effect of Ovary Denervation on the EV-Induced Changes in the Corpora Lutea Size in the Rat

**FIGURE 4:**
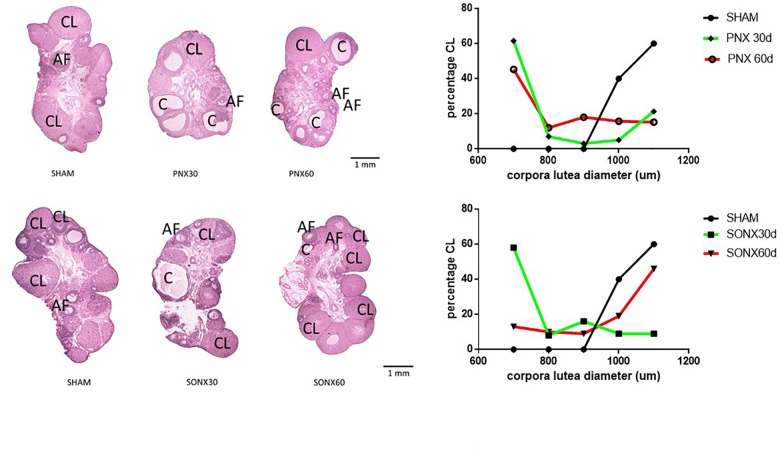
Effect of superior ovarian nerve denervation (SONX30) or 60 (SONX60) days and plexus nerve denervation (PNX30) or 60 (PNX60) days on the morphological aspect of the ovary and size distribution of the CL. Each condition corresponds to the mean number ± SEM of six different rats. CL, corpora lutea; C, cyst; AF, antral follicles.

After the observation of no changes in the CL number, we analyzed the changes in the size of the CL because a larger size means a new CL and a smaller size indicates an older CL. Figure 4 shows a representative picture of the ovary of each experimental condition (left side), sham and denervated ovaries (PNX or SONX). Because of the visual changes found in the ovaries, we performed a distribution of the CL according to the mean size. Ovaries from Sham-operated rats presented a bimodal distribution in which there was an accumulation of new CLs (larger size) and old CLs (smaller size). After 30 days of PNX (Figure 4, up right side) there was no new CL, only old CL. This situation was maintained after 60 days of denervation. On the down right side of Figure 4, Sham ovaries were also presented, and they were compared with the CL after SONX. After 30 days of SONX, only old CL, and no new CL, was detected. However, after 60 days post-denervation a new cohort of CL of larger size appeared, most likely new CL and very similar to the Sham condition, suggesting recovery of ovulation.

### Fertility Studies

The fertility studies were performed in a second experiment in which we used 10 rats for, each treatment group. We did not use rats with PNX because SONX elicited a better ovulatory response according to the previous experiment (defined by the 3Rs of animal use – reduce, refine and replace). In addition the PNX rat had old CL population and hence it represents an infertile rats. Two different periods post-denervation, 30 and 60 days, were used.

In Table 3, we described the general characteristics of the animals during mating and fertilization. Sham-operated rats presented a regular mating behavior – i.e., when the female was exposed to a fertile male during the night of proestrus, 100% presented with the sperm plug the next day, indicating copulation. In the EV group, we observed a sperm plug in only 2 of the 10 rats. The remaining 8 rats accepted the male only after they were exposed to it in a continuous fashion – i.e., they were maintained with the male for 2 weeks and checked daily for the presence of a sperm plug the next morning. It took about 1 week to accept the male and present the sperm plug. Of these 10 rats, only three were pregnant and had live pups. In all the EV-exposed rats that presented implantation points, we observed a lower number of pups per animal (8 in the EV group vs. 12 in the Sham group).

**Table 3:** General characteristics of rats during mating and fertilization.

Condition	n	Spontaneous mating	Induced mating	No. of rats with implantation points	Average No. of implantation points per rat	Average No. pups born per rat
Sham 60 days	10	10	0	10	12	12
EV-90days	10	2	8	3	10	8
EV-60d-SONX30d	10	4	6	1	15	14
EV-90d-SONX60d	10	2	8	7	7	6

SONX during 30 days did not improve the copulatory behavior: 4 of 10 rats presented spontaneous mating. In the 60 days post-SONX group, only 2 of 10 rats demonstrated spontaneous mating.

Despite no change in the copulatory behavior between denervated and non-denervated EV-treated rats, SONX post 60 days of EV (SONX-EV60) presented a higher number of rats with implanted embryos (analyzed after euthanasia and counting the implantation points). This suggests better ovulatory performance and potentially better intrauterine conditions to sustain the pregnancy. This was not analyzed in systematic fashion and remains out of scope for this study.

#### Changes in the Ovarian NE in Relation to a Successful Pregnancy

**FIGURE 5:**
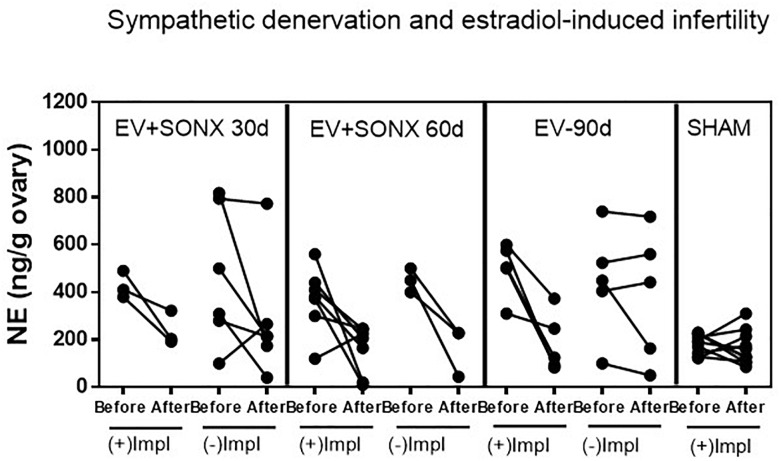
Effect of superior ovarian nerve denervation (SONX) in relation to the pregnancy and implantation points (Impl) in the rat. Because we obtained one ovary during the surgical denervation, we presented the before and after denervation or treatment analysis in the same rat to show the variability in the responses and correlation between NE and pregnancy. Each group corresponded to 10 rats with the exception of the EV+SONX 30d group that contained 9 rats (one of them was euthanized prior due to a rejection to the suture clips after surgery).

To obtain information on a putative relationship of the effectiveness of decrease NE and pregnancy, we show, in Figure 5, the changes in the NE concentration in the ovary before and after denervation for each rat. All sham-operated rats were fertile, and they maintained the ovarian NE concentration in the range of 200 ng/g tissue. After 90 days of the single exposure to EV, there were two groups of rats (Figure 5), three rats were pregnant, and these rats showed a decreased NE concentration in the range of sham rats. The remaining rats presented erratic behavior characterized by no changes in the NE concentration between the beginning of the denervation and end of the protocol. None of these last six rats were pregnant. In the case of pregnancy after 30 days of denervation, most of the rats were infertile, and they also presented erratic behavior regarding the NE concentration. However, in the case of SONX during 60 days, the concentration of NE before and after the surgical procedure was more consistent such that 80% of all of these groups showed a decreased NE. The behavior of the ovarian NE in the groups that were pregnant was consistent with a reduction in the ovarian NE concentration to obtain a concentration near the sham control level.

## Discussion

In this work, we studied the contribution of the PN and SON in the control of ovary function and fertility. Previous evidences suggest that SON denervation disrupt cycling activity in the rat (Forneris and Aguado, 2002). To test the contribution of each, we exposed rats to EV in a protocol that was demonstrated to develop a polycystic ovary phenotype after 60 days of EV exposure (Brawer et al., 1986; Lara et al., 1993; Walters et al., 2012). Additionally, it was been demonstrated that EV exposure induced an increase in the ovarian NE concentration that is correlated with the anovulatory condition induced by EV. Thus, much evidence suggests that NE is a component in infertility associated with EV exposure (Stener-Victorin et al., 2004; Sverrisdottir et al., 2008; Morales-Ledesma et al., 2010). Other studies have demonstrated a developmental window during which the exposure to EV induces irreversible changes in ovary physiology. This period is between 14 and 24 days of age in the Sprague-Dawley rat (Sotomayor-Zarate et al., 2008; Cruz et al., 2012).

In the present work, we found that NE remained increased 30, 60, and 90 days after denervation as we have previously described (Lara et al., 1993; Cruz et al., 2012). Although both PNX and SONX were effective to reduce NE in the ovary in a similar percentage during the first 30 days after the surgical procedure, the values returned to those of EV-treated rats at longer times of denervation. This is likely the same mechanism described in neonatal and adult rats (Lara et al., 1990, 1993, 2000) in which there is a limited time of effect after denervation during which a lower concentration of NE is maintained. This mechanism involves an increase in the intraovarian levels of NGF, and thus regulates the density of innervation in the target organ (Lara et al., 2000). NGF has also been described as an etiological factor in the development of polycystic ovary phenotype in rat and mouse (Dissen et al., 2000; Stener-Victorin et al., 2000; Dissen et al., 2009). Some studies described that unilateral ovariectomy produced a recovery of the cycling activity and in the ovulation of the rats previously treated with EV (Farookhi et al., 1985). The same authors, however, in 1990 described that the rapid ovulation represent an enhanced response of a special precystic population (type III follicles) to the acute procedure and they do not have possibility to produce oocyte (Convery et al., 1990). The other possibility is that unilateral ovariectomy could affect NE innervation as suggested many years ago by Gerendai et al. (1993) and confirmed more recently (Doganay et al., 2010). Some authors have demonstrated that nerve sectioning of SON affect the activity of the contralateral ovary (Morales-Ledesma et al., 2010). A neural central communication to the ovarian sympathetic innervation originates from the paraventricular nucleus of the hypothalamus and its stimulation *in vivo* affects the NE turnover in the ovary (Bernuci et al., 2008; Jara et al., 2010). For this reason and in order to consider this possibility we did the same ULO procedure for the sham denervated rats and compared with this group. In our hands, ULO-sham-operated rats (i.e., non-denervated) used for the pregnancy test (see Figure 5) did not show changes in the ovarian NE concentration even when they were older than 114 days old. Although NE concentration measurement does not demonstrate a regional distribution of nerves, previous evidence obtained using immunohistochemistry techniques have demonstrated that both nerves arrive to different ovarian compartments that are associated with different ovarian functions. PN is associated with the control of blood flow, while SON fibers are associated with follicular development and steroid secretion from the ovary (Lara et al., 2002; Kagitani et al., 2008; Stener-Victorin et al., 2008).

From a physiological perspective, fertility involves both local ovarian hormones and central hypothalamic endocrine control (Plant and Zeleznik, 2006). Sprague-Dawley rats at 4–6 months of age present with regular estrous cycles and optimal fertility (Cruz et al., 2017); hence, they were used for the study in Group 2. The EV effect was demonstrated at 30 and 60 days after exposure. The recovery found at a later age represents the transient effect when EV was administered between 22 and 24 days old (Cruz et al., 2012). The same effect at a later time was verified after SONX or PNX. A clear difference was found, however, 30 days after SONX in which there was an increase in the number of estrous cycles contrary to what occurred in EV-exposed rats and EV-exposed rats plus PNX. SONX appears more effective to recover estrous cyclic activity and, in addition, has more ovulatory events than EV and EV+PNX rats. Thus, it was important to analyze two events related to the PCO phenotype in rats. One of these is cyst formation after EV exposure and the number of CLs as an index of ovulation. In the case of cysts, EV exposure demonstrated a higher number of cysts after 60 days of EV exposure (Walters et al., 2012) but longer times (i.e., when some cyclic activity reappears), the number of cysts was not different from the beginning of treatment, providing more consistency to the partial recovery of estrous cyclicity. However, PNX could not modify the increased number of cysts found by EV treatment. In fact, at longer times – i.e., when estrous cycling activity was recovered – the cysts further increased. Another situation was found by SONX in which there was no increase in the number of cysts by age and, in fact, it was found to decrease by age. Thus, this is another physio-pathological marker suggesting that SONX is more effective than PNX to reverse the PCO phenotype induced by EV. These data have also a direct correlation with the CL number.

In support of a regional effect of NE regarding the origin of the innervation, one of the most important differences was the appearance of the corpora lutea (representing ovulation). SONX could reverse the anovulatory condition induced by EV exposure in the rat in a better way than PNX. This was especially interesting by the appearance of a new cohort of CL of larger size, sustaining the idea of the recovery of the ovulatory process. However, with this analysis, we cannot count the real number of ovulatory points occurring at the ovary and, hence, predict not only the occurrence of ovulation but also the efficiency of the ovulatory process. However, we can predict through this analysis that, 60 days after denervation, rats presented 70% of pregnancy, and 6 of the 7 rats not only were pregnant but also completed pregnancy. In the EV treated SONX-90d rats compared to sham, we observed the number of pups was half of the sham rats (6/rat instead of 12/rat in sham group). PCOS phenotype (EV-90d) had lower number of rats with implanation compared to sham and SONX in EV rats improves the total number of rats with implantation points (3 in EV-90d group vs. 7 in EV-90d-SONX60d group).

Overall, these data strongly support the concept that SON is the principal network of sympathetic fibers arriving to the ovary that are involved in the control of follicular development and ovulation. Most probably, there is a neuronal signal that communicates with the endocrine brain to explain the successful rate of pregnancy found after SONX. These data opens the possibility of the local control of the activity of the SON as a target to regulate ovulation in pathologies affecting follicular development because it was recently suggested using electronic devices regulating the rate of discharge of the nerves (Pikov et al., 2018). In addition, these data project the initial work describing that sympathetic nerve activation is a component in the development of the polycystic ovary phenotype in the rat (Barria et al., 1993; Lara et al., 1993), posterior confirmation by others (Stener-Victorin et al., 2000; Espinoza et al., 2018) and other works suggesting the same etiology for the polycystic ovary phenotype in women (Garcia-Rudaz et al., 1998; Sverrisdottir et al., 2008; Lansdown and Rees, 2012; Ribeiro et al., 2016). Thus, the present work, for the first time, defines the functional role for the different sympathetic networks innervating the rat ovary. These data are important to investigate a similar array of the nerves in the human ovary and to project studies to modify the sympathetic tone by localizing the specific innervation of the follicular compartment in the human ovary.

## Author Contributions

MdC and BP participated in the experimental part of the study, biochemical determination, and follow up of the rats. AS and JW conceived the idea and participated in the study design, data analysis, and manuscript preparation. HL equally contributed to the study design, data collection, analysis, and manuscript preparation.

## Conflict of Interest Statement

AS and JW are employees of Galvani Bioelectronics, a for-profit company. The authors declare that the study was funded by Galvani Bioelectronics and Fondecyt 1170291. HL along with AS and JW (employees of Galvani Bioelectronics) were involved in the design of the study, interpretation of the results. The remaining authors declare that the research was conducted in the absence of any commercial or financial relationships that could be construed as a potential conflict of interest.
